# Right ventricular strain measurements in critically ill patients: an observational SICS sub-study

**DOI:** 10.1186/s13613-022-01064-y

**Published:** 2022-10-03

**Authors:** Madelon E. Vos, Eline G. M. Cox, Maaike R. Schagen, Bart Hiemstra, Adrian Wong, Jacqueline Koeze, Iwan C. C. van der Horst, Renske Wiersema, SICS Study Group

**Affiliations:** 1 University Medical Center Groningen, Department of Anaesthesiology, University of Groningen, Groningen, The Netherlands; 2 University Medical Center Groningen, Department of Critical Care, University of Groningen, Groningen, The Netherlands; 3 Erasmus Medical Center, Department of Internal Medicine, Erasmus University Rotterdam, Rotterdam, The Netherlands; 4 Department of Anaesthesiology, Location VU Medical Center, Amsterdam University Medical Center, Amsterdam, The Netherlands; 5 Department of Critical Care, King’s College Hospital, London, UK; 6 Department of Intensive Care Medicine, University of Maastricht, University Medical Center Maastricht, Maastricht, The Netherlands; 7 Department of Cardiology, Erasmus University Rotterdam, Erasmus Medical Center, Rotterdam, the Netherlands

**Keywords:** Prospective study, Right ventricular function, Strain imaging, Echocardiography, Critical care

## Abstract

**Background:**

Right ventricular (RV) dysfunction is common in critically ill patients and is associated with poor outcomes. RV function is usually evaluated by Tricuspid Annular Plane Systolic Excursion (TAPSE) which can be obtained using critical care echocardiography (CCE). Myocardial deformation imaging, measuring strain, is suitable for advanced RV function assessment and has widely been studied in cardiology. However, it is relatively new for the Intensive Care Unit (ICU) and little is known about RV strain in critically ill patients. Therefore, the objectives of this study were to evaluate the feasibility of RV strain in critically ill patients using tissue-Doppler imaging (TDI) and explore the association between RV strain and conventional CCE measurements representing RV function.

**Methods:**

This is a single-center sub-study of two prospective observational cohorts (Simple Intensive Care Studies (SICS)-I and SICS-II). All acutely admitted adults with an expected ICU stay over 24 h were included. CCE was performed within 24 h of ICU admission. In patients in which CCE was performed, TAPSE, peak systolic velocity at the tricuspid annulus (RV s’) and TDI images were obtained. RV free wall longitudinal strain (RVFWSL) and RV global four-chamber longitudinal strain (RV4CSL) were measured during offline analysis.

**Results:**

A total of 171 patients were included. Feasibility of RVFWSL and RV4CSL was, respectively, 62% and 56% in our population; however, when measurements were performed, intra- and inter-rater reliability based on the intraclass correlation coefficient were good to excellent. RV dysfunction based on TAPSE or RV s’ was found in 56 patients (33%) and 24 patients (14%) had RV dysfunction based on RVFWSL or RV4CSL. In 14 patients (8%), RVFWSL, RV4CSL, or both were reduced, despite conventional RV function measurements being preserved. These patients had significantly higher severity of illness scores. Sensitivity analysis with fractional area change showed similar results.

**Conclusions:**

TDI RV strain imaging in critically ill patients is challenging; however, good-to-excellent reproducibility was shown when measurements were adequately obtained. Future studies are needed to elucidate the diagnostic and prognostic value of RV strain in critically ill patients, especially to outweigh the difficulty and effort of imaging against the clinical value.

**Supplementary Information:**

The online version contains supplementary material available at 10.1186/s13613-022-01064-y.

## Background

In the past years, critical care echocardiography (CCE) has gained interest of clinicians and widespread clinical application for both diagnostics and guidance of treatment in critically ill patients [1]. Right ventricular (RV) dysfunction is often seen in the critically ill, associated with adverse outcomes and increased mortality in various diseases [2, 3]. Therefore, CCE plays an essential role in the non-invasive assessment of RV failure and its impact on hemodynamics [4].

Tricuspid Annular Plane Systolic Excursion (TAPSE) and peak systolic tissue velocity at the tricuspid annulus (RV s’) are frequently used quantitative measurements to evaluate the RV function of which TAPSE is the most reported parameter for RV function in critical care research [5, 6]. Both TAPSE and RV s’ are quick and easy to perform, less dependent on image quality compared to strain, and have good intra- and inter-operator reproducibility. However, both measurements only evaluate longitudinal movement of a fixed site, which can be misleading when perceived to represent global RV function, e.g., in case of regional wall-motion abnormalities [5, 7]. Myocardial deformation imaging assesses regional and global RV function based on strain (Ɛ) measurements. Several studies, in- and outside the intensive care unit (ICU), have shown that strain can detect cardiac dysfunction when conventional measurements are preserved [8–13]. Since RV function is of importance for prognosis, both ultrasound- and ICU papers mention RV strain imaging as an essential clinical research priority, especially in prospective studies with large sample sizes [2, 14].

Several deformation imaging techniques are available to measure strain: colour tissue-Doppler imaging (TDI), measuring TDI-derived strain, and non-Doppler speckle tracking echocardiography (STE), measuring two-dimensional strain or three-dimensional strain [15, 16]. As opposed to STE-derived strain, TDI-derived strain has not been extensively investigated in the critically ill, although both techniques show comparable results in healthy persons and patients with reduced RV function [17].

The clinical application of deformation imaging has widely been studied in cardiology; however, it is relatively new for the ICU and recently published papers mostly focus on patients with sepsis. Vallabhajosyula et al. [18] showed that RV failure is associated with worse 1-year survival in 388 patients with severe sepsis and septic shock [18]. Another study by Orde et al. [8] showed that, in 60 severe sepsis patients, strain can detect cardiac dysfunction when conventional measurements are preserved and that these patients have a higher mortality risk [8]*.* However, little is known about RV strain in unselected cohorts of critically ill patients. Therefore, the primary aim of this study was to evaluate the feasibility of RV strain in an unselected cohort of critically ill patients using TDI. The secondary aim was to explore the association between RV strain and conventional CCE measurements representing RV function.

## Methods

### Design and setting

This was a sub-study of the Simple Intensive Care Studies (SICS)-I and SICS-II, two single-center, prospective observational studies which focused on the prognostic and diagnostic value of combinations of clinical variables in critically ill patients. Details of both studies have been described elsewhere (Clinicaltrials.gov; NCT02912624 and NCT03577405) [19, 20]. The local institutional review board approved both cohort studies (2015/004 and 2018/203).

### Participants

All acutely admitted critically ill patients included within the SICS-I and SICS-II study since the initiation of the sub-study (March 2017) were included [19, 21]. Patients admitted to the ICU of the University Medical Center Groningen (UMCG) were included in the SICS if they were aged 18 or older, had an unplanned ICU admission, and were expected to stay for at least 24 h. Patients were excluded if obtaining research data interfered with clinical care or if patients refused to participate. Additional reasons for exclusion for this sub-study were (1) patients in which no CCE was performed (roughly 50% of SICS-II were included without CCE) due to logistics or physical barriers, i.e., drains or wounds; (2) patients with atrial fibrillation (AF) at examination since strain is sensitive to signal noise and (3) patients in whom no images with an acceptable quality of the RV, according to the predefined requirements (Additional file 1: Section S1), could be obtained.

### Data collection and follow-up

According to our published protocols, all patients underwent a structured clinical examination immediately followed by a protocolized CCE [20]. Patients were included as soon as possible after admission, always within 24 h. Medical research interns and PhD students conducted measurements after focused CCE training by cardiologist-intensivists. Training consisted of self-study on theoretical fundaments, at least two hands-on training sessions on healthy individuals, and finally, at least 20 supervised exams on critically ill. Further details on training have been described elsewhere [22].

CCE was performed transthoracic with the M3S or M4S cardiac transducer of the General Electric Vivid-S6 ultrasound machine. TAPSE and RV s’ were recorded as conventional measures for RV function at the bedside, and all measurements were echocardiogram (ECG)-gated. According to the joint guidelines of the European and American Societies of Echocardiography, a TAPSE below 17 mm and a RV s’ of less than 9.5 cm/s were considered abnormal [23]. Patients were considered to have RV failure when one or both of these measurements were abnormal.

Colour TDI images were obtained from the RV-free wall and the septum in the apical four-chamber view (AP4CH) during five cardiac cycles with a minimal frame rate of 160 frames per second. Using these views, images were obtained to assess RV free wall longitudinal strain (RVFWSL) and RV global four-chamber longitudinal strain (RV4CSL) offline with EchoPAC, version 12.0.1 (General Electric Healthcare, Horten, Norway). An RVFWSL > − 20% and an RV4CSL > − 17%, in other words: less negative, were considered as reduced (Additional file 1: Section S1) [23, 24].

External validation of the CCE measurements was performed by a central independent core laboratory (Groningen Imaging Core Lab, UMCG, Groningen, the Netherlands, www.g-icl.com), a subdivision of the UMCG. In addition, two medical research interns performed offline strain analysis after extensive training by an echocardiography- and strain expert. For further explanation and a detailed protocol on image acquisition and analysis, see Additional file 1: Section S1. To establish intra- and inter-reproducibility, a random sample of 40 cases was reassessed. For sensitivity analysis RV fractional area change (FAC) was measured in a subgroup of patients. Prerequisite was a clear AP4CH view available in the data set making offline analysis possible. A FAC < 35% was considered as reduced [23]. FAC images were not externally validated.

### Statistical analyses

Overall statistical methods were described in the predefined statistical analysis plan of SICS-I [19]. Data were presented as means with standard deviations (SD) when normally distributed or as medians with interquartile ranges (IQR) in case of skewed data. Dichotomous and categorical data were presented in proportions. To assess reproducibility, intraclass correlation coefficient (ICC) (two-way mixed model, the absolute agreement between measurements) was calculated. Based on the 95% confidence interval of the ICC, values were considered as poor (< 0.5), moderate (0.5–0.75), good (0.75–0.9), and excellent (> 0.9) reliability. A two-sided *p* value of ˂ 0.05 was considered statistically significant. Statistical analyses were performed with STATA version 15.0 (StataCorp, College Station, USA).

## Results

**Fig. 1:**
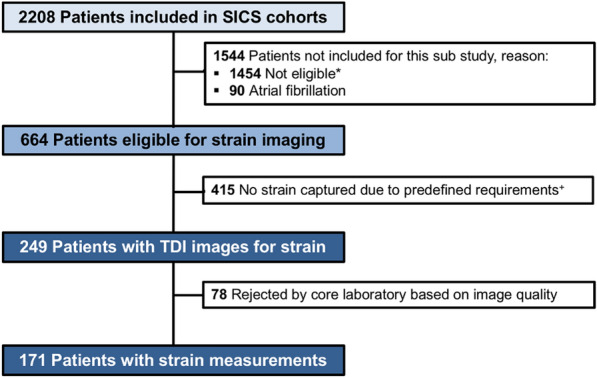
Study flowchart. *Mostly due to inclusion in SICS before sub-study started or not eligible for CCE in SICS-II. ^+^The predefined requirements were: a stable echocardiogram (ECG), an apical four-chamber view (AP4CH) with the myocardial wall clearly visualized and defined, a maximum angle deviation of 15 degrees between the segment of interest and the contraction axis to limit errors caused by angle deviation and a frame rate of at least 160 frames per second for optimal offline strain analysis

Between 27 March 2015 to 22 July 2017 (SICS-I cohort) and 14 March 2018 to 10 July 2019 (SICS-II cohort), 2208 patients were included. 90 patients were excluded due to atrial fibrillation. In total, 664 patients were eligible for the current sub-study (started in March 2017, see Fig. 1). In these patients, TAPSE and RV s’ were available in 526 (79%) and 436 (66%) patients (Additional file 1: Table S1). In 415 of these patients, TDI images were unobtainable at the bedside according to the predefined quality requirements, and in 78 patients, image quality appeared insufficient during offline strain analysis, leaving 171 (26%) patients to be included in this sub-study (Fig. 1). All data represented and discussed below are based on the 171 patients with strain analysis available.

Patient characteristics of the included population are displayed in Table 1. The median time to CCE after ICU admission was 13 h (IQR 6, 18), and at 30 days follow-up, 28 patients (16%) had died. In 122 out of 171 patients (71%), strain measurements of all segments and both TAPSE and RV s’ were available*.* In total, there were 1026 segment measurements measured in 171 patients, of which 57 (6%) segments were missing, leaving RVFWSL to be measured in 155 patients (62%) and RV4CSL in 139 patients (56%) (Additional file 1: Table S2). When strain measurement could be performed, intra- and inter-rater reliability based on the ICC were good to excellent (Table 2).

**Table 1:** Baseline characteristics of included patients

General patient characteristics (*n* = 171)	
Age, years^**#**^	60 [52, 71]
Gender, *n* male (%)	100 (58%)
BMI, kg/cm^2*^	25.2 (4.5)
APACHE IV score^*^	72.5 (26.7)
SAPS-II score^*^	44.1 (15.0)
Admission reason	
Trauma	18 (11%)
Surgical	29 (17%)
Airway problems	4 (2%)
Respiratory insufficiency	33 (19%)
Circulatory insufficiency	12 (7%)
Cardiac, other	6 (3.5%)
Out of hospital cardiac arrest	24 (14%)
Neurological	12 (7%)
Traumatic brain injury	5 (3%)
Sepsis	13 (8%)
Metabolic	6 (3.5%)
Gastro-intestinal	8 (5%)
Clinical variables	
Heart rate, beats per minute^**#**^	80 [68, 91]
Respiratory rate, per minute^**#**^	16 [14, 20]
Systolic blood pressure, mmHg^**#**^	115 [99, 130]
Diastolic blood pressure, mmHg^**#**^	56 [50, 65]
Mean arterial pressure, mmHg^**#**^	74 [67, 85]
Central venous pressure, mmHg^**#**^	8 [5, 12]
Use of vasopressors, *n* (%)	94 (55%)
Use of sedatives, *n* (%)	94 (56%)
Mechanical ventilation, *n* (%)	107 (63%)
PEEP, cm H_2_O^**#**^	7 [5, 8]
Urine output, ml/kg/h^**#**^	0.72 [0.42, 1.21]

*BMI*  Body Mass Index, *APACHE* Acute Physiology and Chronic Health Evaluation, *SAPS*  Simplified Acute Physiology Score*, PEEP*  Positive end expiratory pressure

* Mean ± SD, ^#^median [IQR]

**Table 2:** Obtainability and reproducibility of strain measurements per segment

	RV basal	RV mid	RV apical	S basal	S mid	S apical
Obtainability	99.4% *(170/171)*	93.6% *(160/171)*	98.8% *(169/171)*	93.0% *(159/171)*	92.4% *(158/171)*	89.5% *(153/171)*
Intra-observer ICC (95% CI)	0.90 *(0.80, 0.95)*	0.96 (0.92, 0.98)	0.85 (0.70, 0.92)	0.93 (0.88, 0.97)	0.95 (0.90, 0.97)	0.97 (0.93, 0.98)
Inter-observer ICC (95% CI)	0.92 (0.84, 0.96)	0.93 (0.87, 0.97)	0.78 (0.59, 0.88)	0.87 (0.75, 0.94)	0.88 (0.77, 0.94)	0.90 (0.79, 0.95)

*RV* Right ventricle free wall, *S* Septal wall, *ICC* Intraclass Correlation Coefficient, *CI* Confidence Interval

*Estimated average time for offline manual strain analysis: 15 min per patient.*

### Conventional RV CCE measurements and TDI RV strain

**Fig. 2:**
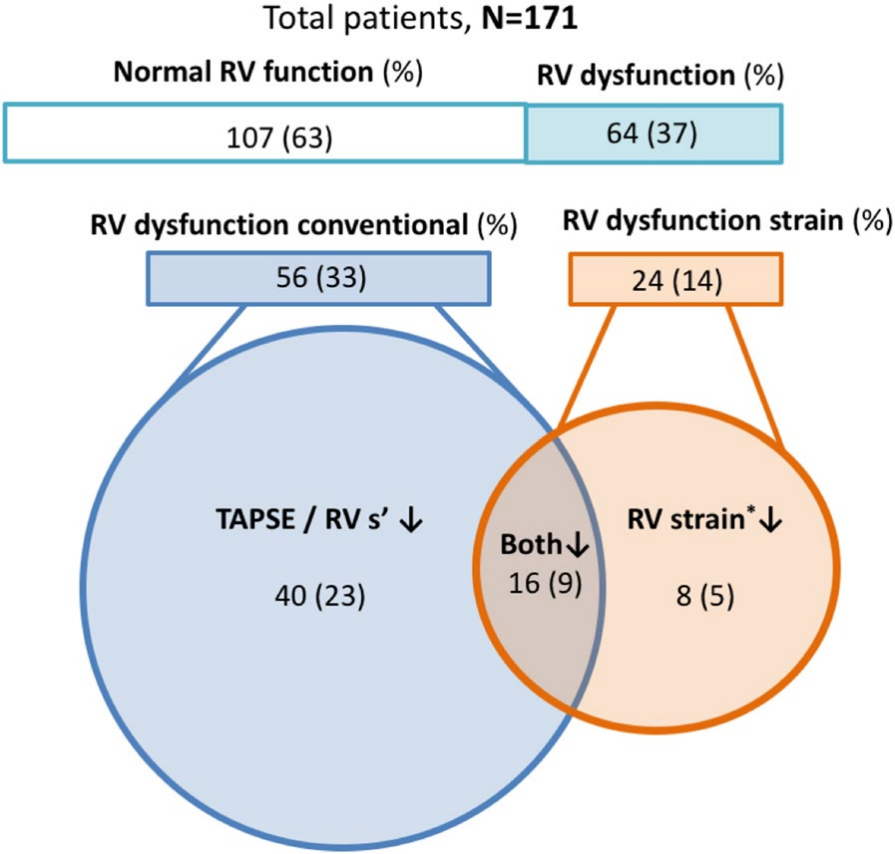
Venn diagram conventional RV CCE measurements and RV strain indicating RV dysfunction. *Indicating TDI-derived RV global longitudinal peak strain (RV4CSL) and RV free wall longitudinal peak strain (RVFWSL)

All echocardiography variables are displayed in Table 3. The overall incidence of RV dysfunction in these SICS cohorts based on conventional analysis was 33% (56 patients). In 20 patients (12%), both TAPSE and RV s’ were reduced. Twenty-four patients (14%) had RV dysfunction based on RVFWSL or RV4CSL. In 40 patients (23%), TAPSE or RV s’ indicated RV dysfunction, whereas RVFWSL or RV4CSL were preserved. Figure 2 shows the visual data distribution between groups indicating RV dysfunction. In 14 patients (8%), RVFWSL, RV4CSL, or both were reduced, whereas conventional RV function measurements were preserved. These patients had a significantly higher acute physiology and chronic health evaluation (APACHE) IV score and a higher simplified acute physiology score (SAPS II). In addition, they received more frequent vasoactive medication and showed higher 30-day mortality rates (OR 4.1 95% CI 1.2–13.9; p 0.026) (Additional file 1: Table S3). Sensitivity analysis with RV FAC was performed in a subgroup of patients (*n* = 60). Median FAC was 41% [28, 32] and FAC was reduced in 20 patients (33%). Distribution of RV dysfunction based on FAC versus strain was similar to TAPSE/RV s’ versus strain (Additional file 1: Figure S1). In 2 patients (3%) RVFWSL and RV4CSL were reduced, whereas FAC was preserved. These patients showed similar characteristics with significantly higher APACHE IV and SAPS II scores; however, no significant difference in 30-day mortality was observed (*p* = 0.055) (Additional file 1: Table S5).

**Table 3:** Echocardiography variables

	*N* = 171
Cardiac output, L/min^#^	4.90 [3.91, 5.6.45]
Cardiac index, L/min/m^2#^	2.58 [2.01, 3.21]
TAPSE, mm^*^	20.05 (± 5.95)
RV s’, cm/s ^#^	12.4 [10.00, 15.30]
Strain RV free wall, (%)	
Basal^*^	− 28.77 (± 8.76)
Mid^*^	− 27.07 (± 9.29)
Apical^#^	− 24.07 [− 31.00, − 17.97]
Strain septum, (%)	
Basal^*^	− 23.07 (± 7.40)
Mid^*^	− 19.40 (± 6.33)
Apical^#^	− 18.72 [− 22.26, − 13.53]
RV4CSL, (%)^*^	− 23.90 (± 5.52)
RVFWSL, (%)^*^	− 27.11 (± 7.22)

*Mean ± SD, ^#^median [IQR]

*TAPSE* Tricuspid annular plane systolic excursion, *RV s*´  right ventricular systolic excursion, *RV4CSL*  RV global longitudinal peak strain, *RVFWSL*  RV free wall longitudinal peak strain

TAPSE, RV s’, FAC, RVFWSL and RV4CSL were all significantly lower in mechanically ventilated patients compared to the non-ventilated patients (Additional file 1: Table S4). A high level of PEEP (> 8) did not show any differences between groups.

## Discussion

In this prospective observational study, we assessed the feasibility of RV strain measurements using TDI in an unselected population of critically ill patients. Although image acquisition was challenging, when TDI images were obtainable according to the predefined rules, strain analysis was possible with good-to-excellent consistency between repeated measurements. Furthermore, when comparing RVFWSL and RV4CSL to conventional RV CCE measurements, strain indicated RV dysfunction in a small subgroup, where conventional RV function measurements were normal.

To our knowledge, prospective data on TDI-derived RV strain in an unselected cohort of critically ill patients of this size is not available yet. Previous studies on strain in critically ill patients are in general not in unselected cohorts, not TDI focused and included, on average, sixty cases [8, 25]. This may be related to the limited possibilities of obtaining appropriate echo-cardiogenic windows and subsequent poor image quality in critically ill patients. In addition, suboptimal positioning, physical barriers, and mechanical ventilation combined with the thin RV wall and its position behind the sternum make RV imaging even more complicated [26]. This, combined with the fact that high image quality is essential for TDI analysis to be performed explains why many patients were excluded.

Various circumstances influence RV function and subsequently potentially RV strain. As with all CCE measurements, RV strain is operator-dependent. Compared to a recently published paper of global and segmental longitudinal RV strain measurements, we observed less variability based on the intra- and inter-observer ICCs [27]. Another important modifier of RV function frequently applied in critically ill patients is mechanical ventilation. Two studies that evaluated STE strain suggest that non-invasive ventilation acutely decreases STE-derived RV strain (and thus decreases RV function) in patients with obstructive sleep apnea syndrome, and increasing PEEP decreases STE-derived LV strain in mechanically ventilated patients [28, 29]. Mechanical ventilation leads to reduced venous return and increased RV afterload, which in turn can lead to pressure overload and systolic RV dysfunction [30]. Indeed, mechanically ventilated patients in this study had significantly lower global, free wall, and segmental RV strain, but this seemed independent of PEEP. We also established in our cohort that TAPSE, RV s’ and FAC were also significantly reduced in mechanically ventilated patients (Additional file 1: Table S4). Since the majority of our cohort (63%) received mechanical ventilation, loading conditions secondary to mechanical ventilation might have influenced our TAPSE and RV s’ measurements and this might explain the unexpected finding that conventional RV function measurements indicated RV dysfunction more often in our cohort than stain. We corrected in advance for technical factors, such as angle dependency, frame rate, and Doppler gain, but we could not correct for the influence of respiration and the explanation given above remains a hypothesis. When future studies evaluate the prognostic value of RV strain, awareness of the possible impact of mechanical ventilation on RV function is important.

RV strain indicated RV dysfunction in a small subgroup (*n* = 14) when conventional RV function measurements were preserved. This finding was irrespective of the amount of PEEP that was applied. This subgroup had higher severity of illness scores, and we observed a significant higher 30-day mortality in these patients. Sensitivity analysis comparing FAC with RV strain provided similar data distribution and results.

Several studies have already shown the prognostic value of both left ventricular (LV) and RV STE-derived strain [8–11]. In an unselected cohort of 64 ICU patients, Nafati et al. found that the feasibility of 2D LV strain measurement in an unselected cohort was 77% and their data suggests that strain could identify early LV dysfunction [25]. The feasibility rate of Nafati et al. cannot be directly compared to our cohort, since their study focused on the LV, which is usually easier to image due to the anatomical position of the LV and a thicker free wall. Moreover, their population had lower SAPS II scores and was less frequently supported by mechanical ventilation of vasopressor therapy. Another study in 57 patients with pulmonary hypertension and RV dysfunction showed that RVFWLS provided important prognostic information when TAPSE was preserved [31]. Our data were not powered to conclude that strain suggests subtle RV dysfunction in this cohort and it was a single measurement. Future studies should include repeated measurements to evaluate the evolution of strain and conventional measures of RV function over time.

## Strengths and limitations

This is the first study evaluating TDI-derived RV strain in a relatively large prospective unselected cohort of critically ill patients. In addition, it is a cohort with detailed patient characteristics and images were validated and measured by an independent expert. Even though this study was initiated before the PRICES recommendations by Huang, Sanfilippo and colleagues were published, we incorporated most of the preferred items and methodology [32, 33]. However, several limitations must be acknowledged. First, we included 171 (26%) out of the 664 eligible patients, emphasizing the limitations of CCE related research in critically ill patients due to suboptimal positioning and clinical interference. Nevertheless, we report real-world experience and show the challenge of obtaining strain in critically ill patients, which is also an important clinical finding. We hypothesize that even when taking extra time and additional expertise, obtainability will remain low in acute critically ill patients. Second, we used colour TDI-derived strain, while nowadays non-Doppler speckle tracking echocardiography (STE) strain is more frequently applied in clinical practice. We realize that this is an important limitation; however, this was a methodological choice for the following reasons; our data acquisition started in 2017 and at that time the choice to use TDI images was mainly based on general echocardiography image quality of the first part of the SICS-I cohort. Our echocardiography and strain expert estimated that the obtainability of AP4CH views with clear 360-degree wall delineation for STE would be much lower than the obtainability of wall focused views for TDI. Another important consideration was the effect of tachyarrhythmias on the reliability of TDI and STE measurements. Lord et al. [34] showed in healthy athletes that reliability of STE strain measurements reduces in case of tachycardia due to a greater data variability, while TDI measurements remain unchanged [34]. Based on the incidence of tachyarrhythmias in the general ICU population, our previous SICS-I cohort (before start of this sub-study) and the risk of under sampling in case of STE and tachyarrhythmias we decided to use colour TDI measurements in our sub-study [35]. Although the choice for TDI can be retrospectively questioned, we have collected data that comply with state-of-the-art papers mentioning the research priority of advanced echo techniques (e.g., strain) to identify RV failure before onset [2]. Third, the images for RV strain were obtained by trained researchers under the supervision of cardiologist-intensivists. Thus, there might have been patients in whom experts would have been able to obtain an image of acceptable quality. Fourth, the image acquisition followed by transportation to an external ultrasound-lab and offline manual analysis was a time-consuming process limiting clinical applicability. Estimated average time for offline manual strain analysis in our study was 15 min per patient (reassessment for reproducibility excluded). However, nowadays, several vendors offer software packages that enable reliable bedside CCE analysis, but we are unaware of studies using these techniques for RV strain in critically ill patients. Last, we conducted a single-center study. Collaboration with other centers and other ICUs will increase generalizability.

## Implications

Our results must be interpreted as explorative data in critical care, and further investigation is needed to unravel the exact diagnostic and prognostic value of RV strain assessments in the critically ill [18, 36]. In the future, strain calculation based on images with a lower quality may be possible due to developed techniques and artificial intelligence.

## Conclusions

TDI RV strain imaging in critically ill patients is challenging; however, good-to-excellent reproducibility was shown when measurements were adequately obtained. In a small subgroup strain indicated RV dysfunction when conventional RV measurements were preserved. This subgroup was characterized by higher severity of illness scores. Future studies are needed to elucidate the diagnostic and prognostic value of RV strain in critically ill patients, especially to outweigh the difficulty and effort of imaging against the clinical value.

## Supplementary Information

**Additional file 1: Table S1. **Overview of eligible patients from the SICS cohorts and RV function measurements obtained. **Table S2**. Overview of missing strain segments. **Table S3.** Baseline characteristics based on conventional RV function measurements preserved and strain reduced (CPSR). **Table S4.** RV echocardiography variables categorized by receiving mechanical ventilation (with peep > 8). **Table S5. **Baseline characteristics sensitivity analysis: RV fractional area change (FAC) preserved versus RV strain reduced (FPSR). **Figure S1. **Sensitivity analysis; Venn diagram FAC versus RV Strain indicating RV dysfunction. **Section S1. **Detailed protocol image acquisition and strain analysis.

## Data Availability

The data set used and/or analyzed during the current study is available from the corresponding author on reasonable request.

## Abbreviations

APACHE: Acute physiology and chronic health evaluation
AP4CH: Apical four chamber view
AP5CH: Apical five chamber view
CCE: Critical care echocardiography
CI: Confidence Interval
CPR: Cardiopulmonary resuscitation
CPSR: Conventional RV function measurements (TAPSE/RV s’) preserved and strain reduced
ECG: Echocardiogram
Ɛ: Strain
FAC: Fractional area change
ICC: Intraclass Correlation Coefficient
ICU: Intensive Care Unit
IQR: Interquartile range
LV: Left ventricle
MV*: Mechanical ventilation
PEEP: Positive end expiratory pressure
RV: Right ventricle
RV4CSL: RV global longitudinal peak strain
RVFWSL: RV free wall longitudinal peak strain
RV s’: Peak systolic tissue velocity at the tricuspid annulus
SAPS: Simplified acute physiology score
SD: Standard deviation
SICS: Simple intensive care studies
STE: Non-Doppler speckle tracking echocardiography
TAPSE: Tricuspid annular plane systolic excursion
TDI: Tissue doppler imaging
UMCG: University Medical Center Groningen
