# Supplementary material for: Right ventricular strain measurements in critically ill patients: an observational SICS sub-study
Source: Ann Intensive Care. 2022 Oct 3;12:92. doi: 10.1186/s13613-022-01064-y (PMC9530097; doi:10.1186/s13613-022-01064-y)
Supplement: Supplementary file 1 — Additional file 1: Table S1. Overview of eligible patients from the SICS cohorts and RV function measurements obtained. Table S2. Overview of missing strain segments. Table S3. Baseline characteristics based on conventional RV function measurements preserved and strain reduced (CPSR). Table S4. RV echocardiography variables categorized by receiving mechanical ventilation (with peep > 8). Table S5. Baseline characteristics sensitivity analysis: RV fractional area change (FAC) preserved versus RV strain reduced (FPSR). Figure S1. Sensitivity analysis; Venn diagram FAC versus RV Strain indicating RV dysfunction. Section S1. Detailed protocol image acquisition and strain analysis. [file 13613_2022_1064_MOESM1_ESM.docx]

**Right ventricular strain measurements in critically ill patients: an observational SICS sub-study, Vos et al.**

**INDEX**

**Table S1.** Overview of eligible patients from the SICS cohorts and RV function measurements obtained.

**Table S2**. Overview of missing strain segments

**Table S3.** Baseline characteristics based on conventional RV function measurements preserved and strain reduced (CPSR).

**Table S4.** RV echocardiography variables categorized by receiving mechanical ventilation (with peep > 8).

**Table S5.** Baseline characteristics sensitivity analysis: RV fractional area change (FAC) preserved versus RV strain reduced (FPSR).

**Figure S1.** Sensitivity analysis; Venn diagram FAC versus RV Strain indicating RV dysfunction

**Section S1.** Detailed protocol image acquisition and strain analysis

**REFERENCES**

**TABLES**

**Table S1.** Overview of eligible patients from the SICS cohorts and RV function measurements obtained.

| N = | SICS I & II cohort  (n = 664) | SICS I cohort  (n = 293) | SICS II cohort  (n = 371) |
| --- | --- | --- | --- |
| **TAPSE** | 526 (79,2%) | 256 (87,4%) | 270 (72,8%) |
| **RV S’** | 436 (65,7%) | 239 (81,6%) | 197 (53,1%) |
| **RV strain** | 171 (25,6%) | 88 (30,0%) | 83 (22,4%) |

Abbreviations: *TAPSE* = Tricuspid annular plane systolic excursion, *RV s*´ = Right ventricular systolic excursion, *RV* = right ventricular.

**Table S2.** Overview of missing strain segments

| **Myocardial wall** | **Segment** | **Missing, n (%)** | **Reason (n = x)** |
| --- | --- | --- | --- |
| RV free wall | Basal | 1 (0.6%) | Inadequate imaging (n=1) |
|  | Mid | 11 (6.4%) | Inadequate imaging (n=8), Unreliable strain curve (n=3) |
|  | Apical | 2 (1.2%) | Inadequate imaging (n=2) |
| Septum | Basal | 12 (7%) | Inadequate imaging (n=5), Unreliable strain curve (n=2), AP5CH (n=5) |
|  | Mid | 13 (7.6%) | Inadequate imaging (n=4), Unreliable strain curve (n=4), AP5CH (n=5) |
|  | Apical | 18 (10.5%) | Inadequate imaging (n=7), Unreliable strain curve (n=5), AP5CH (n=5) |

Abbreviations: *AP5CH* = apical five chamber view.

**Table S3.** Baseline characteristics based on conventional RV function measurements preserved and strain reduced (CPSR).

|  | **No CPSR**  **(n = 108)** | **CPSR**  **(n = 14)** | **p-value** |
| --- | --- | --- | --- |
| Age, years ^#^ | 58.5 [50.5, 69.0] | 62.0 [58.0, 66.0] | 0.62 |
| Gender, n male (%) | 62 (57.4%) | 11 (78.6%) | 0.13 |
| BMI, kg/cm^2 *^ | 24.89 (4.17) | 24.12 (4.50) | 0.52 |
| APACHE IV score ^*^ | 68.22 (26.74) | 86.29 (27.87) | *0.021* |
| SAPS-II score ^*^ | 41.83 (14.40) | 53.57 (15.01) | *0.005* |
| Heart rate beats per minute ^#^ | 80.0 [67.5, 90.0] | 82.0 [65.0, 99.0] | 0.86 |
| Respiratory rate, per minute ^#^ | 16.0 [14.0, 20.0] | 16.0 [12.0, 21.0] | 0.62 |
| Mechanical ventilation, n (%) | 66 (61.1%) | 12 (85.7%) | 0.071 |
| Use of vasopressors, n (%) | 54 (50.0%) | 11 (78.6%) | *0.044* |
| Use of sedatives, n (%) | 55 (51.4%) | 11 (78.6%) | 0.055 |
| 30-day mortality | 13 (12%) | 5 (36%) | *0.019* |

* = Mean ± SD. ^#^ = median [IQR]. Abbreviations: *CPSR* = Conventional RV function measurements (TAPSE / RV s’) preserved and strain reduced, *BMI* = Body Mass Index, *APACHE* = Acute Physiology and Chronic Health Evaluation, *SAPS* = Simplified Acute Physiology Score*.*

**Table S4.**  RV echocardiography variables categorized by receiving mechanical ventilation and PEEP > 8.

| **Strain variables** | **Not mechanically ventilated**  **(n = 67)** | **Mechanically ventilated**  **(n = 104)** | **p-value** | **PEEP ≤ 8**  **(n = 85)** | **PEEP > 8**  **(n = 19)** | **p-value** |
| --- | --- | --- | --- | --- | --- | --- |
| TAPSE (mm)*  RV S’ (cm/s)^#^  FAC^#^ (%) *n=30  Strain RV free wall, (%)   - Basal* - Mid* - Apical^#^   Strain Septum, (%)   - Basal* - Mid* - Apical^#^   RV4CSL, (%)*  RVFWSL, (%)* | 21.81 (± 5.59)  15.00 [12.00, 17.00]  46.00 [38.00, 51.00]  -30.39 (± 8.77)  -29.88 (± 8.93)  -26.01 [-31.72, -21.49]  -23.90 (± 8.45)  -19.90 (± 5.48)  -21.44 [-24.46, -14.98]  -25.49 (± 4.85)  -29.25 (± 6.33) | 18.92 (± 5.93)  11.00 [9.30, 14.00]  38.00 [32.00, 25.00]  -27.72 (± 8.66)  -25.26 (± 9.11)  -22.41 [-30.02, -16,16]  -22.55 (± 6.66)  -19.08 (± 6.83)  -17.49 [-21.06, -12.68]  -22.89 (± 5.71)  -25.69 (± 7.44) | *0.002*  *<0.001*  *0.031*  0.052  *0.002*  *0.011*  0.26  0.43  *<0.001*  *0.006*  *0.002* | 18.98 (± 6.15)  11.00 [9.33, 14.00]  38.00 [32.00, 46.00]  -27.13 (± 7.90)  -25.92 (± 9.03)  -22.52 [-31.63, -15.82]  -23.15 (± 6.39)  -19.46 (± 6.90)  -17.51 [-20.81, -12.68]  -23.20 (± 5.71)  -25.94 (± 7.55) | 18.66 (± 4.99)  11.00 [8.00, 14.00]  34.00 [26.00, 42.00]  -30.33 (± 11.33)  -21.032 (± 8.87)  -22.30 [-25.73, -17.26]  -19.88 (± 7.37)  -17.15 (± 6.29)  -14.23 [-21.06, -12.76]  -20.29 (± 5.25)  -23.78 (± 6.65) | 0.84  0.79  0.48  0.15  0.071  0.66  0.059  0.22  0.87  0.15  0.37 |

* = Mean ± SD. ^#^ = median [IQR]. Abbreviations: *TAPSE* = Tricuspid annular plane systolic excursion, *RV s*´ = Right ventricular systolic excursion, *FAC* = fractional area change, *RV4CSL* = RV global longitudinal peak strain, *RVFWSL*= RV free wall longitudinal peak strain, *PEEP* = positive end-expiratory pressure (cmH2O)

**Table S5.** Baseline characteristics sensitivity analysis: RV fractional area change (FAC) preserved versus RV strain reduced (FPSR).

|  | **No FPSR**  **(n = 58)** | **FPSR**  **(n = 2)** | **p-value** |
| --- | --- | --- | --- |
| Age, years ^#^ | 59.0 [52.0, 68.0] | 69.5 [66.0, 73.0] | 0.27 |
| Gender, n male (%) | 31 (53%) | 2 (100%) | 0.19 |
| BMI, kg/cm^2 *^ | 25.1 (4.5) | 22.9 (42.2) | 0.48 |
| APACHE IV score ^*^ | 71.6 (26.2) | 114.5 (65.8) | *0.036* |
| SAPS-II score ^*^ | 44.1 (14.6) | 69.00 (31.1) | *0.025* |
| Heart rate beats per minute ^#^ | 80.5 [71.0, 97.0] | 89.0 [76.0, 102.0] | 0.62 |
| Respiratory rate, per minute ^#^ | 17.0 [14.0, 20.0] | 21.5 [16.0, 27.0] | 0.40 |
| Mechanical ventilation, n (%) | 35 (60%) | 2 (100%) | 0.26 |
| Use of vasopressors, n (%) | 26 (45%) | 2 (100%) | 0.12 |
| Use of sedatives, n (%) | 29 (51%) | 2 (100%) | 0.17 |
| 30-day mortality | 5 (9%) | 1 (50%) | 0.055 |

* = Mean ± SD. ^#^ = median [IQR]. Abbreviations: *FPSR* = RV fractional area change preserved and strain reduced, *BMI* = Body Mass Index, *APACHE* = Acute Physiology and Chronic Health Evaluation, *SAPS* = Simplified Acute Physiology Score*.* This sub analysis was based on unvalidated images.

**Figure S1.** Sensitivity analysis; Venn diagram FAC versus RV Strain indicating RV dysfunction

**
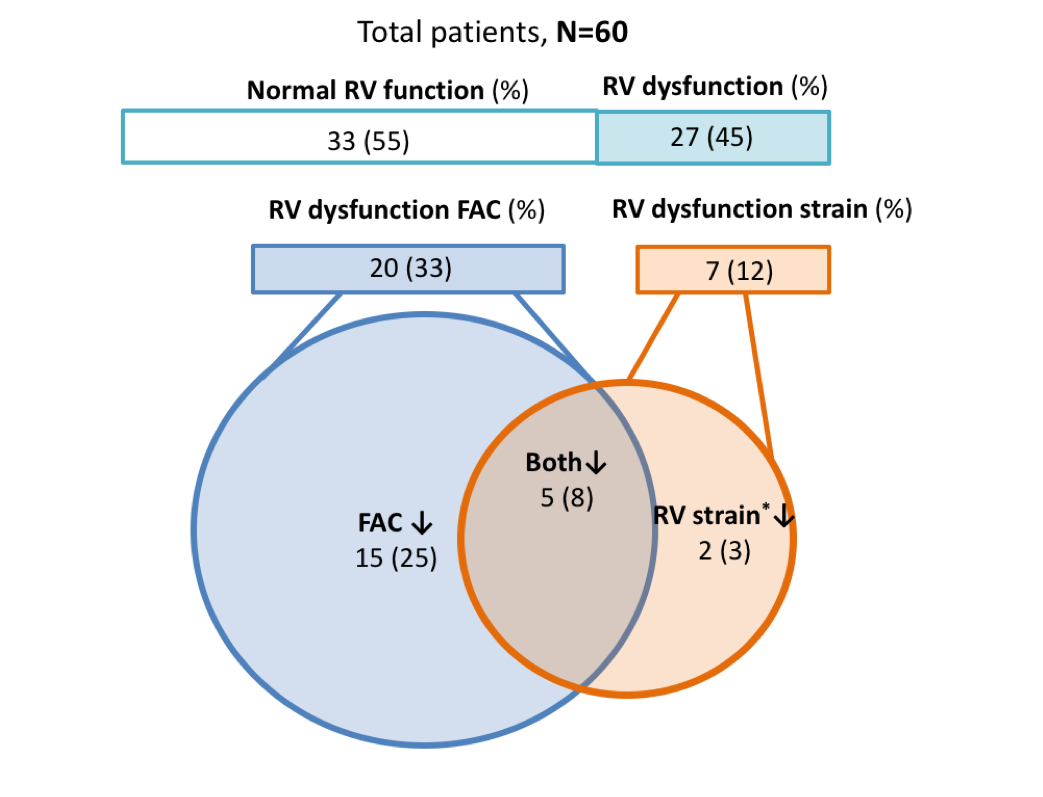
**

* Indicating TDI derived RV global longitudinal peak strain (*RV4CSL) and* RV free wall longitudinal peak strain (*RVFWSL)*. Abbreviations: FAC = RV Fraction area of change.

**SECTION**

**Section S1. Detailed protocol image acquisition and strain analysis**

**Colour tissue Doppler imaging**

Critical care echocardiography (CCE) was performed with the General Electric Vivid-S6 mobile ultrasonography machine, using the M3S of M4S cardiac transducer. Color TDI images were obtained from the RV free wall and the septum in the apical four-chamber view (AP4CH) during five cardiac cycles. Conditions that had to be met before imaging was a stable echocardiogram (ECG) and a clear AP4CH view, where all myocardial walls were clearly visualised and defined. Acquisition started with standard 2D mode for optimal orientation. To limit errors caused by angle deviation, the segment of interest was positioned in the centre of the ultrasound window to align the myocardial wall motion to the centre with a maximum accepted deviation of 10 to 15 degrees from the contraction axis. For optimal offline strain analysis, a high frame rate of at least 160 frames per second was required. To meet this requirement, the window was narrowed.

**Strain analysis**

Recorded images were transferred to a central independent core laboratory (Groningen Imaging Core Lab, UMCG, Groningen, the Netherlands, www.gicl.com) for anonymisation and offline analysis. All recorded images were validated in a stepwise manner. Strain analysis was performed with EchoPAC, version 12.0.1 (General Electric Healthcare, Horten, Norway) by two medical research interns (MV and MS) after training by a strain-specialised echocardiography technician. Longitudinal peak strain was measured with no distinction between peak- and post-systolic strain. The RV free wall and the septum were divided into three segments according to the task force recommendations, corresponding with the basal, mid, and apical segments of the investigated wall [1]. A region of interest (ROI) was positioned in each segment and tracked during the recorded cardiac cycles. The exact position of the ROI within the full wall segment was based on the most optimal shape of the longitudinal strain curve [2]. For each ROI, the three most reliable strain curves were selected based on morphology and the absence of artefacts. If possible, longitudinal peak strain was measured for each segment in three consecutive cardiac cycles, and measurements of the different ROIs were aimed to conduct in the same cardiac cycle. However, an optimal strain curve morphology was chosen over consecutiveness. To reduce signal noise, the longitudinal peak strain of each ROI was measured at the peak value of three selected curves and averaged. The ROI was not measured and considered missing if no reliable strain curve could be obtained due to artefacts, as shown in E-table *1.* Baseline drift was applied.

RV free wall longitudinal peak strain (RVFWSL) was calculated as the average strain of the RV free wall segments. Global longitudinal RV peak strain (RV4CSL) was calculated as the average of both the RV free wall - and the septum segments. Based on the guidelines for echocardiography assessment of the right heart, RVFWSL > -20% (in other words: less negative) was considered as a reduced [3]. In literature, no consensus value is known for RV4CSL, however, the lowest expected value in 238 healthy volunteers was -17% [4]. Therefore, we considered an RV4CSL > -17% (in other words: less negative) as reduced. Vendor – and technique differences were taken into account while selecting these cut-off values with an existing lack of proper (TDI) RV strain reference values in critically ill patients. [5–7]

**REFERENCES**

1. Badano LP, Kolias TJ, Muraru D, Abraham TP, Aurigemma G, Edvardsen T, et al. Standardization of left atrial, right ventricular, and right atrial deformation imaging using two-dimensional speckle tracking echocardiography: a consensus document of the EACVI/ASE/Industry Task Force to standardize deformation imaging. Eur Hear J - Cardiovasc Imaging. 2018;19:591–600.

2. Teske AJ, De Boeck BW, Melman PG, Sieswerda GT, Doevendans PA, Cramer MJ. Echocardiographic quantification of myocardial function using tissue deformation imaging, a guide to image acquisition and analysis using tissue Doppler and speckle tracking. Cardiovasc Ultrasound. 2007;5:27.

3. Lang RM, Badano LP, Mor-Avi V, Afilalo J, Armstrong A, Ernande L, et al. Recommendations for Cardiac Chamber Quantification by Echocardiography in Adults: An Update from the American Society of Echocardiography and the European Association of Cardiovascular Imaging. Eur Hear J – Cardiovasc Imaging. Oxford University Press; 2015;16:233–71.

4. Morris DA, Krisper M, Nakatani S, Köhncke C, Otsuji Y, Belyavskiy E, et al. Normal range and usefulness of right ventricular systolic strain to detect subtle right ventricular systolic abnormalities in patients with heart failure: a multicentre study. Eur Hear J – Cardiovasc Imaging. 2017;18:212–23.

5. Mirea O, Pagourelias ED, Duchenne J, Bogaert J, Thomas JD, Badano LP, et al. Intervendor Differences in the Accuracy of Detecting Regional Functional Abnormalities. JACC Cardiovasc Imaging. 2018;11:25–34.

6. Longobardo L, Suma V, Jain R, Carerj S, Zito C, Zwicke DL, et al. Role of Two-Dimensional Speckle-Tracking Echocardiography Strain in the Assessment of Right Ventricular Systolic Function and Comparison with Conventional Parameters. J Am Soc Echocardiogr. 2017;30:937-946.e6.

7. Teske AJ, De Boeck BWL, Olimulder M, Prakken NH, Doevendans PAF, Cramer MJ. Echocardiographic Assessment of Regional Right Ventricular Function: A Head-to-head Comparison Between 2-Dimensional and Tissue Doppler–derived Strain Analysis. J Am Soc Echocardiogr. 2008;21:275–83.
